# B Vitamins, Homocysteine and Bone Health

**DOI:** 10.3390/nu7042176

**Published:** 2015-03-30

**Authors:** Valentina Fratoni, Maria Luisa Brandi

**Affiliations:** Department of Surgery and Traslational Medicine, University of Florence, Viale Pieraccini, 6-50139 Florence, Italy; E-Mail: vale.frat@gmail.com

**Keywords:** B Vitamins, folate, homocysteine, plasma total homocysteine (tHcy), methylene tetrahydrofolate reductase (MTHFR), MTHFR 677C_T polymorphism, fracture, bone

## Abstract

Nutrition is one of the most important modifiable factors involved in the development and maintenance of good bone health. Calcium and Vitamin D have confirmed and established roles in the maintenance of proper bone health. However, other nutritional factors could also be implicated. This review will explore the emerging evidence of the supporting role of certain B Vitamins as modifiable factors associated with bone health. Individuals with high levels of homocysteine (hcy) exhibit reduced bone mineral density (BMD), alteration in microarchitecture and increased bone fragility. The pathophysiology caused by high serum homocysteine is not completely clear regarding fractures, but it may involve factors, such as bone mineral density, bone turnover, bone blood flow and collagen cross-linking. It is uncertain whether supplementation with B Vitamins, such as folate, Vitamin B1, and Vitamin B6, could decrease hip fracture incidence, but the results of further clinical trials should be awaited before a conclusion is drawn.

## 1. Introduction

**Figure 1:**
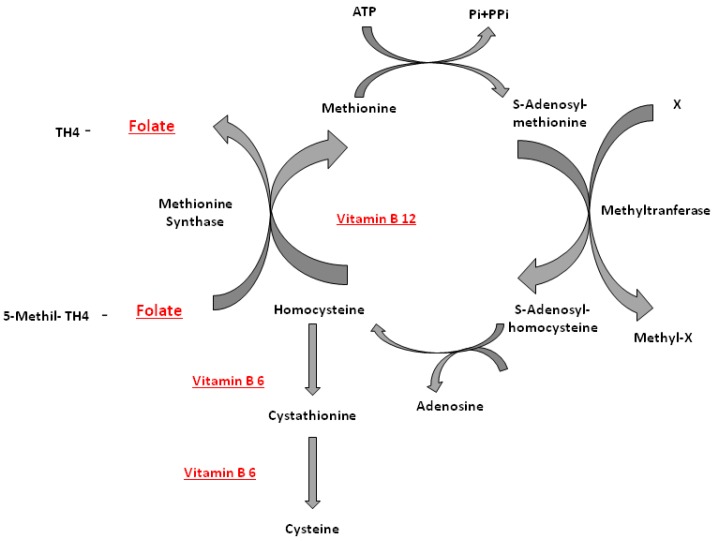
Homocysteine metabolism: B12 (Vitamin B12), B6 (vitaminb6), MTHFR (methylene tetrahydrofolate reductase).

Many factors contribute to bone health. Bone is a dynamic tissue in a constant state of remodeling. Bone formation exceeds bone resorption generally in the first three decades of life, the age when peak bone mass is achieved. After this time, bone resorption is favored, and bone loss ensues [1]. Osteoporosis is a chronic, multifactorial disorder characterized by low bone mass and microarchitectural deterioration of bone tissue [2,3]. Deterioration of bone quality predisposes affected individuals to an increased risk of fragility fracture [1,4]. The most common osteoporotic fracture sites are the spine, hip and wrist, with both spine and hip fractures accompanied by considerable disability and increased morbidity and mortality [1,5], in addition to increased social and economic burden [3]. This burden is expected to increase substantially in Europe in the coming decades due to a rise in life expectancy [6]. Combined supplementation of calcium and Vitamin D have been proven to reduce bone loss and fracture incidence [7]. However, it is possible that nutritional factors not typically linked with bone health could play a protective role for bone. Association studies have identified vitamins related to fractures or bone mineral density [8]. Emerging evidence in groups of healthy individuals suggests a protective association of certain B Vitamins, in particular Vitamin B12 and folic acid, a detrimental effect of homocysteine and the 677C_T polymorphism in the gene encoding the folate metabolizing methylene tetrahydrofolate reductase (MTHFR) enzyme [3,9]. High concentrations of homocysteine and low levels of Vitamin B12 and folate, the main determinants in the metabolism of homocysteine [10,11], have been associated with low bone mineral density (BMD) and a higher risk of fractures in the elderly [3]. Analyses of randomized controlled trials have shown that supplementation of folic acid (0.5–5 mg day^−1^) has resulted in reducing the levels of homocysteine in blood up to 25%; the co-supplementation of folic acid and Vitamin B12 (0.5–5 mg day^−1^ and 500 mcg day^−^^1^, respectively) provided a further reduction of 7% with a decrease in serum total homocysteine by 32% [12]. To date, the mechanisms linking homocysteine to increased fracture risk have not yet been clarified. It is known that serum homocysteine is regulated by Vitamin B12 and folic acid, and supplementation with these vitamins decreases serum homocysteine levels. It is also known that folate, Vitamin B12, Vitamin B6 and riboflavin are involved in the metabolism of an S-containing amino acid, homocysteine. Homocysteine metabolism links the methionine cycle with the folate cycle. A first link between homocysteine (hcy) and the skeleton has been noted in studies of hyperhomocysteinuria, a metabolic disorder characterized by exceedingly high levels of hcy in the plasma and urine. Individuals with hyperhomocysteinuria exhibit numerous skeletal defects, including reduced BMD and osteopenia [13]. Homocysteine comes from the breakdown of methionine, one of the essential amino acids used for protein synthesis. Homocysteine can be converted to cystathionine with Vitamin B6 and further to cysteine. Alternatively, homocysteine can be remethylated to methionine with help from vitamin B12 [8]. The latter reaction is catalyzed by methionine synthase and requires 5-methyltetrahydrofolate, the principal circulating form of folate, and Vitamin B12 in its co-factor form, methylcobalamin. The formation of 5-methyltetrahydrofolate is catalyzed by the MTHFR enzyme [1] (Figure 1).

The pathway for the conversion of homocysteine to methionine is the transfer of a methyl group from 5-methyltetrahydrofolate to homocysteine, catalyzed by the Vitamin B12-dependent enzyme, methionine synthase [8]. Reducing elevated homocysteine levels through folic acid and Vitamin B12 supplementation could theoretically ensure proper bone health and prevent osteoporosis. However, at present, no consensus has been reached on the magnitude of the association between Vitamin B12, folate, homocysteine and bone health, nor on the possible effect of Vitamin B12 and folate supplementation on bone health [3].

## 2. Nutrition and B Vitamins

The B Vitamins are thiamine (B1), riboflavin (B2), niacin (B3, also called nicotinamide or nicotinic acid amide), pantothenic acid (B5), pyridoxine (B6), biotin (B7), folic acid or folate (B9) and cobalamin (B12). B Vitamins play an important role in growth, development and other bodily functions by promoting enzymatic activity. Food sources of Vitamin B are both plant and animal. Vitamin B deficiency can lead to the emergence of many diseases. An understanding of how a deficiency in B Vitamins influences the health of the population could lead to targeted prevention for the benefit of public health. Governments all over the world have therefore started recommending levels of daily intake of Vitamin B in order to keep the population healthy. Therefore, recommended levels, known as recommended dietary allowance (RDA) and tolerable upper intake level (UL), have been established. In addition to calcium and Vitamin D, which are already well known, B Vitamin (especially Vitamin B6, B9 and B12) are also involved in bone health [14].

A healthy diet can provide all the B Vitamin necessary to keep the body healthy, easily reaching the recommended dietary intakes. However, many people have an unbalanced diet, low in healthy foods (fruits, vegetables, meat, fish, cheese, eggs, legumes and cereals), meaning that they do not get the recommended amounts. Especially in older persons, those with the highest fracture risk, intake is often a problem, and more importantly, the absorption of Vitamin B12 is reduced, leading to Vitamin B12 deficiencies in the elderly. In fact, some B Vitamin can be obtained from vegetable foods, while others (such as Vitamin B12) can be obtained only from animal products [14]. B Vitamin are water-soluble, meaning that any excess intake is largely excreted in the urine. Supplements containing B Vitamin are generally thought to be safe, but still should not be taken in very large doses. Possible side effects can vary, depending on which B vitamin is taken [15].

Table 1 lists the recommended dietary allowance (RDA) for B Vitamin important for bone health. It also lists the foods richest in Vitamin B6, Vitamin B12 and folic acid according to the National Nutrient Database for Standard Reference of the United States Department of Agriculture (USDA).

**Table 1:** RDAs (recommended dietary allowances and adequate intakes) for micronutrients.

VIT.B	RDA	UL (≥19 years)	Food	Serving	mcg
**Vitamin B6**	Men: 1.3 mg day^−1^ (19–50 years), 1.7 mg day^−1^ (>50 years) Women: 1.3 mg day^−1^ (19–50 years), 1.5 mg day^−1^ (>50 years)	Men and Women: 100 mg day^−1^	Fortified breakfast cereal	1 cup	0.5–2.5
			Salmon, wild (cooked)	3 ounces *	0.48–0.80
			Potato, Russet, with skin (baked)	1 medium	0.70
			Turkey, light meat (cooked)	3 ounces	0.69
			Avocado	1 medium	0.52
			Chicken, light meat without skin (cooked)	3 ounces	0.51
			Spinach (cooked)	1 cup	0.44
			Banana	1 medium	0.43
			Dried plums, pitted	1 cup	0.36
			Banana	1 medium	0.43
			Hazelnuts (dry roasted)	1 ounce	0.18
			Vegetable juice cocktail	6 ounces	0.13
**folic acid**	Men and women: 400 μg day^−1^	Men and women: 1000 μg day^−1^	Fortified breakfast cereal	1 cup	200–400
			Orange juice (from concentrate)	6 ounces	83
			Spinach (cooked)	1/2 cup	132
			Asparagus (cooked)	1/2 cup (~6 spears)	134
			Lentils (cooked)	1/2 cup	179
			Garbanzo beans (cooked)	1/2 cup	141
			Lima beans (cooked)	1/2 cup	78
			Bread	1 slice	20 (folic acid) ^§^
			Pasta (cooked)	1 cup	60 (folic acid) ^§^
			Rice (cooked)	1 cup	60 (folic acid) ^§^
**Vitamin B12**	Men and women: 2.4 μg day^−1^	ND	Clams (steamed)	3 ounces	84.1
			Mussels (steamed)	3 ounces	20.4
			Mackerel (Atlantic, cooked, dry-heat)	3 ounces *	16.1
			Crab (Alaska king, steamed)	3 ounces	9.8
			Beef (lean, plate steak, cooked, grilled)	3 ounces	6.9
			Salmon (chinook, cooked, dry-heat)	3 ounces	2.4
			Rockfish (cooked, dry-heat)	3 ounces	1.0
			Milk (skim)	8 ounces	0.9
			Turkey (cooked, roasted)	3 ounces	0.8
			Brie (cheese)	1 ounce	0.5
			Egg (poached)	1 large	0.4
			Chicken (light meat, cooked, roasted)	0 ounces	0.3

UL: tolerable upper intake level, established by the Food and Nutrition Board of the U.S. Institute of Medicine, the UL is the highest level of daily intake of a specific nutrient likely to pose no risk of adverse health effects in almost all individuals of a specified age; ND: no date, list of the nutrient content of specific foods according to the USDA National Nutrient Database for Standard Reference; * A three-ounce serving of meat or fish is about the size of a deck of cards; ^§^ To increase the revenue of folic acid in the population, the Food and Drug Administration (FDA) required the addition of 1.4 milligrams (mg) of folic acid per kilogram (kg) of grain to be added to refined grain products, which are already enriched with niacin, thiamin, riboflavin and iron, as of 1 January 1998.

### 2.1. Vitamin B6

#### 2.1.1. Food Sources

Humans cannot synthesize Vitamin B6, and therefore, it must be obtained from the diet. Vitamin B6 is found in a variety of foods, including fish, poultry, nuts, legumes, potatoes and bananas. The analysis of data collected in the NHANES 2003–2004 showed that, in the United States, Vitamin B6 intake from food is estimated to be an average of 1.9 mg day^−1^ [16]. However, despite the fact that these values are well above those indicated by the RDA, we know that the serum levels of Vitamin B6 were low for all age groups. This phenomenon could be explained by the fact that many plant foods contain a unique form of Vitamin B6, called pyridoxine glucoside; this form of Vitamin B6 appears to be only about half as bioavailable as Vitamin B6 from other food sources or supplements [17]. Vitamin B6 in a mixed diet has been found to be approximately 75% bioavailable [13].

#### 2.1.2. Supplements

Those who follow a very strict vegetarian diet might need to increase their Vitamin B6 intake by eating foods fortified with Vitamin B6 or by taking a supplement. Doses of Vitamin B6 above 200 mg were associated with cancer and neurotoxicity [18]. Currently, the relationship between this Vitamin and cases of colorectal cancer are still under discussion [19]; the relationship between excess Vitamin B6 and neuropathy is more clear [18,20].

### 2.2. Folic Acid

#### 2.2.1. Food Sources

Green leafy vegetables are foods especially rich in folate. Table 1 shows the main sources of folate based on the content of folic acid in micrograms (mcg). Citrus fruits, legumes and fortified cereals are also good sources of folate [13]. Blood homocysteine levels have decreased since the FDA mandated folic acid fortification of grain supply [21].

#### 2.2.2. Supplements

The principal form of supplementary folate is folic acid. It is available in single ingredient and combination products, such as B-complex Vitamin and multivitamins. The fortification of flour foods [22] has markedly reduced the incidence of birth defects, especially those of the central nervous system, such as dysfunction of the neural tube (DTN), anencephaly and spina bifida [23,24], and other types of skull malformations and heart defects [25,26]. Woman who decide to conceive are strongly recommended to take 400 mcg (0.4 mg) of folic acid per day to prevent the onset of these serious birth defects [23]. A folic acid supplementation ≥0.4 mg day^−1^ seems to be associated with an increased risk of cancer [27], but the role of folate is complex and still not clear. It might depend on the dose and timing of supplementation during carcinogenesis. It is thought to protect against the initiation of cancer, while it may enhance growth and progression. The risk of toxicity from folic acid is low, because folate is a water-soluble vitamin and is regularly removed from the body through urine [28], so although the risk of intakes >1000 μg day^−1^ (the FDA’s safe upper limit of daily intake) would be minimal, the actual effect of folate intake has yet to be determined [29].

### 2.3. Vitamin B12

#### 2.3.1. Food Sources

Vitamin B12 is found only in foods of animal origin [30]. Foods that are high in Vitamin B12 include liver (26–58 μg (100 g)^−1^), beef and lamb (1–3 μg (100 g)^−1^), chicken (trace-1 μg (100 g)^−1^), eggs (1–2.5 μg (100 g)^−1^) and dairy foods (0.3–2.4 μg (100 g)^−1^). There are no naturally occurring bioactive forms of Vitamin B12 from plant sources. Some plant foods contain added Vitamin B12. Some foods that are contaminated or fermented by bacteria, e.g., tempeh and Thai fish sauce, have been reported to contain Vitamin B12 [31], although these may be poorly absorbed. In normal humans, the absorption of Vitamin B12 from foods has been shown to vary depending on the quantity and type of protein consumed [32].

#### 2.3.2. Supplements

Most people have no trouble getting the RDA of 2.4 mcg day^−1^ of Vitamin B12 from food. However, those who regularly consume a diet free of cheese, milk, dairy products and eggs may have a deficiency of this vitamin at any age [33]. The Vitamin B12 deficiency can be caused by malabsorption, a not vary or inadequate diet (especially in the elderly), by prolonged vegetarian or vegan diet or ovo-lacto diet. Furthermore, pregnant and/or lactating women following vegetarian or vegan diets are at high risk of deficiency due to the increased metabolic demand for Vitamin B12. Together with B Vitamin-fortified foods and supplements, these foods may constitute new alternatives to prevent Vitamin B12 deficiency in individuals consuming vegetarian diets. Furthermore, due to the increased probability of food-bound Vitamin B12 malabsorption with increasing age, it would be a good habit to set a nutritional program that includes the intake of Vitamin B12 through supplements or fortified foods (such as fortified cereals) [15]. Deficiency of Vitamin B12, though often undiagnosed, may affect a significant number of people, especially older adults. One symptom of Vitamin B12 deficiency is megaloblastic anemia, which is indistinguishable from that associated with folate deficiency. Large doses of folic acid given to an individual with an undiagnosed Vitamin B12 deficiency could correct megaloblastic anemia without correcting the underlying Vitamin B12 deficiency, leaving the individual at risk of developing irreversible neurologic damage [34].

The neurologic symptoms of Vitamin B12 deficiency include: numbness and tingling of the hands and, more commonly, the feet; difficulty walking; memory loss; disorientation; and dementia with or without mood changes. Although the progression of neurologic complications is generally gradual, such symptoms may not be reversed with treatment of Vitamin B12 deficiency, especially if they have been present for a long time. Neurologic complications are not always associated with megaloblastic anemia and are the only clinical symptom of Vitamin B12 deficiency in about 25% of cases [15]. Although Vitamin B12 deficiency is known to damage the myelin sheath covering cranial, spinal and peripheral nerves, the biochemical processes leading to neurological damage in Vitamin B12 deficiency are not yet fully understood [35].

Because Vitamin B12 is a water soluble vitamin, it is difficult to overdose or build up Vitamin B12 toxicity. With water-soluble vitamins, the body excretes excess amounts in the urine, rather than storing them [36]. Because of the low toxicity of Vitamin B12, no tolerable upper intake level (UL) has been set by the U.S. Food and Nutrition Board [15]. While there are not very many side effects related to the high intake of Vitamin B12, excess supplementation may be associated with prostate cancer [37].

## 3. Bone, B Vitamins and Homocysteine

Our skeleton is not an inert structure, but an active organ, made up of tissue and cells in a continual state of activity throughout a lifetime. The majority of bone mass is acquired during the growth phase of bone development [38,39]. The maximum amount of bone acquired is known as peak bone mass (PBM) [38,40]. Sixty to 80% of PBM is determined by genetics, while the remaining 20%–40% is influenced by lifestyle factors, primarily nutrition and physical activity [41]. It is important to achieve maximum PBM at a young age (before 30 years of age) in order to protect the skeleton against progressive bone loss associated with aging. From the moment the loss of bone tissue begins, the occurrence of osteoporosis with a high risk for fractures of the hip and spine becomes possible [42]. The World Health Organization (WHO) defines osteopenia as the phase before osteoporosis; osteoporosis is a condition of increased bone fragility and susceptibility to fracture due to loss of bone mass. BMD, expressed as the amount of mineral present for given area/volume of bone, is a convenient clinical marker for evaluating bone mass and is associated with osteoporotic fracture risk [43]. In addition to BMD [39], there are other modifiable risk factors that play a role in the risk of osteoporotic fractures. Risk factors are generally cumulative [44] and include microarchitecture and bone geometry, balance, mobility, muscle strength and environmental factors. Bone microarchitecture, in addition to bone density, is also important for determining the status of bone health. A lower number of trabeculae and increased trabecular separation may represent one of the parameters to assess the risk of fractures. The evaluation of texture parameters, such as trabecular bone score (TBS) or average Hurst parameter (H), in addition to BMD and risk factors related to bone fragility, could help find, in groups of non-osteoporotic people, those who are at high risk of fractures [45]. Osteoporosis is a leading cause of morbidity and mortality in the elderly [46]. Regarding the prevalence of osteoporosis in Europe, it is estimated that by the year 2050, the number of men and women to be affected will be more than 30 million [47]. Osteoporosis influences quality of life, as well as life expectancy [48], because the major consequence of osteoporosis is fractures, and hip fractures are especially associated with institutionalization and increased mortality. In the year 2000, approximately nine million fractures occurred worldwide, leading to a loss of 5.8 million disability adjusted life-years (DALYs) [49]. Due to a rise in life expectancy, the economic burden of osteoporotic fractures in Europe is expected to increase substantially in the coming decades. Osteoporotic subjects represent a group of individuals at risk, and for this reason, there is growing interest among scientists and clinicians to search for specific nutrients capable of ensuring good bone health and preventing disease-related skeletal complications [50]. Until now, calcium and Vitamin D have been considered the primary objective of nutritional prevention of osteoporosis. Based on this review of the literature, it has emerged that other nutrients are also needed to maintain healthy bone. In particular, although more studies are needed, the proper intake of B Vitamin might improve bone health [51]. There are many hypotheses that explain the potential role of B Vitamins and/or homocysteine in bone health [1].

*In vitro* studies have shown that slightly elevated concentrations of homocysteine (from 10 mmol L^−1^) increase osteoclast activity and bone resorption [52,53], leading in turn to an inhibitory effect of homocysteine on bone formation [54]. Kang and Trelstad found that homocysteine interfered with collagen cross-links from purified rat skin collagen [1]. Additional *in vitro* studies indicate that high concentrations of Hcy inhibit the activity of lysyl oxidase (an enzyme involved in cross-linking of collagen) and thereby stimulate osteoclast activity in elevated concentration [55,56,57,58]. Interference in cross-link formation would cause an altered bone matrix, resulting in more fragile bones [59].

Animal studies have shown that a deficiency in folic acid and Vitamins B6 and B12 can lead to increased levels of homocysteine and, consequently, an increased production of free radicals and oxidative stress, which lead to endothelial dysfunction [60,61], decreased bone blood flow and, eventually, osteoporosis [62]. Decreased bone blood flow would intuitively lead to bone disease from lack of nutrient delivery to the bone. However, these investigations do not demonstrate the causality between Hcy and changes in bone blood flow, and they do not provide any data regarding the mechanisms behind their observations [60]. Clemens *et al.* observed that mice deficient in the synthesis of Vitamin B12 have growth retardation and a comparative scarcity of osteoblasts. Evidence emerging *in vivo* suggests that Vitamin B12 may interfere with growth hormone signaling in these mice and exert its downstream effects on osteoblasts [63]. The Roman Garcia study has shown that Vitamin B12 deficiency in a genetic mouse model results in severe growth retardation post-weaning and osteoporosis, and the severity and time of onset of this phenotype in the offspring depends on the genotype of the mother. The study shows how Vitamin B12 plays a vital role by regulating positively post-weaning growth and bone formation through the synthesis of taurine, thus paving the way for potential therapies to increase bone mass [64]. Recently, increased plasma homocysteine (HCY) has been suggested to be an independent risk factor for osteoporotic fractures in elderly persons [2,65]. Little is known, however, about the mechanistic role of Hcy in osteoporosis. A link between Hcy and bone disease was first made in 1966, when McKusick [66,67] hypothesized a disturbed collagen cross-linking in patients with homocystinuria.

Holstein *et al.* have demonstrated that high concentrations of homocysteine and S-adenosylhomocysteine in bone, as well as a low capacity of methylation, concern a morphology of reduced bone in humans [68]. Starting from *in vitro* [69] and *in vivo* data [70], they have concluded that there is an association between the altered bone morphology and high bone concentrations of homocysteine and S-adenosylhomocysteine, but not between bone morphology and impaired methylation ability [68]. The effect of subtle elevations of plasma hcy on bone health is difficult to demonstrate in human studies, as they are related to mixed results [13,15]. Some report an association between elevated plasma hcy and fracture risks [2,65,71], while others find no relationship [72,73,74]. However, it is not clear whether this is related to tHcy *by itself*, to the level of Vitamins B12, B6 or folate, which are required for its metabolism, or to other causes of elevated tHcy, such as environmental factors or underlying disease [75]. A recent meta-analysis of observational studies has found that elevated homocysteine levels and low vitamin B12 and folate levels have been associated with structural deterioration of bone tissue, and Vitamin B6 has been found to be deficient in people with hip fractures [3]. In another study, which examined older men and women, it has been shown that subjects who showed low plasma concentrations of Vitamin B6 (<20 nmol L^−1^) are associated with an altered morphology of human bone [76] and had a higher average annual bone loss than those with normal B6 concentrations [77]. It is also important to highlight that a study to investigate the effect of Vitamin B12 on osteoblast-related proteins showed that levels of osteocalcin and alkaline phosphatase skeletal blood were lower in patients with Vitamin B12 deficiency. The results suggested that the activity of osteoblasts depends on Vitamin B12 and that bone metabolism is affected by Vitamin B12 deficiency, but still, it is not known whether the deficiency of Vitamin B12 produces clinically-significant bone disease [78]. The assessment of the association of Hcy and Vitamin B12 status and the combined effect of the two with broadband ultrasound attenuation (BUA), bone turnover markers and fracture on a group of subjects of the Longitudinal Aging Study Amsterdam led to the conclusion that high Hcy and low concentrations of Vitamin B12 were significantly associated with low BUA, high bone turnover markers and an increased risk of fracture [79].

Currently, there are few studies designed to evaluate the effect of folic acid and Vitamin B12 on lowering plasma homocysteine and the effects on bone health [80].

The Rotterdam study, regarding osteoporosis, BMD and the onset of vertebral fractures, showed that increased levels of homocysteine are a factor of strong and independent risk for osteoporotic fractures [2,81]. A related study, which has examined the association between intake of Hcy-related B Vitamin (riboflavin, pyridoxine, folate and Vitamin B12) and femoral neck bone mineral density (FN-BMD) and the risk of fracture in a large population-based cohort of elderly Caucasians concluded that increased dietary riboflavin and pyridoxine intake was associated with higher FN-BMD [82]. In a controlled clinical trial, Reynolds *et al*. studied the correlation between fracture risk and the lack of Vitamin B6, in particular of pyridoxal-5’-phosphate (PLP), the most important form of Vitamin B6 present in human serum, concluding that PLP can be a causative factor for hip fracture under its role in the activity of a key regulatory protein [83].

In the Hordaland Homocysteine Study [84], folate has been linked to BMD and a reduced fracture risk [85], but there is limited evidence to support a direct mechanistic effect of folate on bone.

In the Heart Outcomes Prevention Evaluation (HOPE) 2 of the trial, the effects of five years of vitamin supplementation (2.5 mg of folic acid, 50 mg of vitamin B 6 and 1 mg of Vitamin B 12 per day) on fractures were evaluated [86]. What emerged was that although the plasma levels of Hcy were reduced in the treatment group, there were no significant differences between treatment and placebo on the incidence of skeletal fractures [87]. The B-PROOF study (B Vitamins for the Prevention of Osteoporotic Fracture), a randomized controlled trial, tried to determine whether Vitamin B12 and folic acid supplementation reduces osteoporotic fracture incidence in hyperhomocysteinemic elderly individuals [88,89]. Data from this study show that combined Vitamin B12 and folic acid supplementation had no effect on osteoporotic fracture incidence in an elderly population. Since the treatment was also associated with a higher incidence of cancer, Vitamin B12 plus folic acid supplementation cannot be recommended at present for fracture prevention in elderly people [89]. The Vitatopos trial seems to confirm that treatment with B Vitamins had no effect on the incidence of osteoporotic fractures in treated patients [90]. The population studies can lead to inconsistencies in the results obtained due to both differences in study populations [59,91,92] and to heterogeneity between studies. The heterogeneity may be explained by the differences in the mean age of the study populations, differences in the mean status of Vitamin B12, folate and homocysteine, differences in the sex distribution of the study population and the duration of follow-up [3]. One randomized controlled trial, designed to determine in healthy elderly subjects whether lowering homocysteine with B Vitamins affects plasma biomarkers of bone turnover, led to the conclusion that supplementation with folic acid and Vitamins B-6 and B-12 lowered plasma homocysteine, but had no beneficial effect on bone turnover estimated from biomarkers of bone formation and resorption [93]. In a two-year prospective, placebo-controlled, double-blind trial, Sato *et al.* [94] observed a strong reduction in fracture incidence in stroke patients who received high doses of folate and vitamin B12. In addition, low cobalamin status has been shown to reduce osteoblast activity [78].

### Genetic Studies

Several epidemiological studies have been performed investigating the relationship between MTHFR 677C_T polymorphism in the gene that encodes for the MTHFR enzyme and bone health and fracture risk [3,9]. Methylene reductase physiologically catalyzes the conversion of 5,10-methylene-5-methylene, which is used for the methylation of homocysteine to methionine, thereby decreasing the concentration of homocysteine [8]. The 677C_T polymorphism in the MTHFR gene is widely reported as the most common genetic determinant of hyperhomocysteinemia (>15 μmol L^−1^) [95,96] and low erythrocyte folate concentrations [96,97]. Mendelian randomization studies show that homozygosity for the variant allele (TT genotype), which occurs at a rate of 10% in most ethnic groups, leads to 25% higher tHcy concentrations than occur in individuals with the common genotype (CC) [98]. The variant enzyme develops a greater propensity to dissociate from the FAD. Riboflavin (Vitamin B2) in the form of FAD acts as a cofactor for MTHFR. The supplementation with low-dose riboflavin could stabilize MTHFR activity *in vivo* in homozygous individuals [99]. The 677C_T polymorphism was associated with BMD at all measured sites, with a 23% increased risk for all fractures in individuals with the MTHFR 677TT genotype compared with those with the CT or CC genotypes [9,100]. The Aberdeen Osteoporosis Screening Study showed that a low intake of riboflavin (vitamin B2) in subjects with the TT genotype had a negative effect on the BMD of the femoral neck [101]. Studies have reported that individuals with the TT genotype and low folate levels (<9 nmol L^−1^) had a lower BMD than those with CC or CT genotypes at the same concentration of plasma folate, suggesting an important gene-nutrient interaction [102]. In support of these findings, a Danish Osteoporosis Prevention Study has concluded that individuals with the TT genotype had a significantly reduced BMD with low dietary intakes associated with various B Vitamins, including folic acid, B12, B6 and riboflavin [103]. Such findings provide evidence to support a detrimental effect of the polymorphism combined with low B Vitamin intakes on robust bone health outcomes (*i.e*., BMD and fracture risk) and indicate that B Vitamins may have the potential to modulate any negative effect of this polymorphism on bone health [1]. Many studies are designed to evaluate the association between the presence of MTHFR 667C_ T and the incidence of fractures and BMD, but often, the results were inconsistent. This trend could be explained by the different levels of plasma folate taken into account [8]. Factors such as differences in ethnicity (considerable variation in the frequency of this polymorphism between populations) and variation in the age of the populations investigated probably also contribute to inconsistencies among studies [1].

## 4. Conclusions

There are still conflicting data regarding the relationship between high levels of homocysteine, low concentrations of B Vitamins and low bone mineral density. This epidemiological evidence is further reinforced by genetic studies that show an association between the common MTHFR 677C_T polymorphism and the risk of osteoporosis. Certainly, mechanisms related to changes in collagen cross-linking may lead to a change in bone structure.

Epidemiological cohort studies, however, show strong associations between low levels of Vitamin B12 and homocysteine serum concentrations and a high incidence of fractures.

Additional studies are needed to show how low levels of Vitamin B are causally associated with the risk of osteoporosis and whether there are benefits given by the supplementation of Vitamin B for bone health.

## References

[1] Clarke M., Ward M., Strain J.J., Hoey L., Dickey W., McNulty H. (2014). B-vitamins and bone in health and disease: The current evidence. Proc. Nutr. Soc..

[2] Van Meurs J.B.J., Dhonukshe-Rutten R.A.M., Pluijm S.M.F., van der Klift M., de Jonge R., Lindemans J. (2004). Homocysteine levels and the risk of osteoporotic fracture. N. Engl. J. Med..

[3] Van Wijngaarden J.P., Doets E.L., Szczecińska A., Souverein O.W., Duffy M.E., Dullemeijer C., Cavelaars A.E., Pietruszka B., Van’t Veer P., Brzozowska A. (2013). Vitamin B12, folate, homocysteine, and bone health in adults and elderly people: A systematicreview with meta-analyses. Nutr. Metable.

[4] Scott J.M. (1997). Bioavailability of vitamin B12. Eur. J. Clin. Nutr..

[5] Carmel R., Green R., Rosenblatt D.S., Watkins D. (2003). Update on cobalamin, folate, and homocysteine. Hematol. Am. Soc. Hematol. Educ. Program..

[6] Kanis J.A., Johnell O. (2005). Requirements for DXA for themanagement of osteoporosis in Europe. Osteoporos. Int..

[7] Tang B.M.P., Eslick G.D., Nowson C. (2007). Use of calcium or calcium in combination with vitamin D supplementationto prevent fractures and bone loss in people aged 50 years and older: A meta-analysis. Lancet.

[8] Swart K.M., van Schoor N.M., Lips P. (2013). Vitamin B12, folic acid, and bone. Curr. Osteoporos. Rep..

[9] Wang H., Liu C. (2012). Association of MTHFR C667T polymorphism with bone mineral density and fracture risk: An updated meta-analysis. Osteoporos. Int..

[10] Selhub J., Jacques P.F., Wilson P.W.F., Rush D., Rosenberg I.H. (1993). Vitamin status and intake as primary determinants of homocysteinemia in an elderly population. J. Am. Med. Assoc..

[11] Jacques P.F., Bostom A.G., Wilson P.W.F., Rich S., Rosenberg I.H., Selhub J. (2001). Determinants of plasma total homocysteine concentration in the Framingham Offspring cohort. Am. J. Clin. Nutr..

[12] Robert C. (1998). Lowering blood homocysteine with folic acid based supplements: Meta-analysis of randomised trials. Homocysteine Lowering Trialists’ Collaboration. BMJ.

[13] Herrmann M., Widmann T., Herrmann W. (2005). Homocysteine—A newly recognised risk factor for osteoporosis. Clin. Chem. Lab. Med..

[14] American Cancer Society Vitamin B Complex. http://www.cancer.org/treatment/treatmentsandsideeffects/complementaryandalternativemedicine/herbsvitaminsandminerals/vitamin-b-complex.

[15] Food and Nutrition Board, Institute of Medicine (1998). Dietary Reference Intakes for Thiamin, Riboflavin, Niacin, Vitamin B6, Folate, Vitamin B12, Pantothenic Acid, Biotin, and Choline.

[16] Morris M.S., Picciano M.F., Jacques P.F., Selhub J. (2008). Plasma pyridoxal 5’-phosphate in the US population: The National Health and Nutrition Examination Survey, 2003–2004. Am. J. Clin. Nutr..

[17] Clayton P.T. (2006). B6-responsive disorders: A model of vitamin dependency. J. Inherit. Metab. Dis..

[18] Schaumburg H., Kaplan J., Windebank A., Vick N., Rasmus S., Pleasure D., Brown M.J. (1983). Sensory neuropathy from pyridoxine abuse. A new megavitamin syndrome. N. Engl. J. Med..

[19] Zhang X.H., Ma J., Smith-Warner S.A., Lee J.E., Giovannucci E. (2013). Vitamin B6 and colorectal cancer: Current evidence and future directions. World J. Gastroenterol..

[20] Renwick A.G. (2006). Toxicology of micronutrients: Adverse effects and uncertainty. J. Nutr..

[21] Quinlivan E.P., McPartlin J., McNulty H. (2002). Importance of both folic acid and vitamin B12 in reduction of risk of vascular disease. Lancet.

[22] Berry R.J., Bailey L., Mulinare J., Bower C. (2010). Fortification of flour with folic acid. Food Nutr. Bull..

[23] Williams J., Mai C.T., Mulinare J., Isenburg J., Flood T.J., Ethen M., Frohnert B., Kirby R.S. (2015). Updated estimates of neural tube defects prevented by mandatory folic Acid fortification-United States, 1995–2011. Morb. Mortal. Wkly. Rep..

[24] Pitkin R.M. (2007). Folate and neural tube defects. Am. J. Clin. Nutr..

[25] Lupo P.J., Goldmuntz E., Mitchell L.E. (2010). Gene-gene interactions in the folate metabolic pathway and the risk of conotruncal heart defects. J. Biomed. Biotechnol..

[26] Antony A.C. (2007). In utero physiology: Role of folic acid in nutrient delivery and fetal development. Am. J. Clin. Nutr..

[27] Wien T.N., Pike E., Wisløff T., Staff A., Smeland S., Klemp M. (2012). Cancer risk with folic acid supplements: A systematic review and meta-analysis. BMJ Open.

[28] Food and Agriculture Organization (2002). Human Vitamin and Mineral Requirements.

[29] Quinlivan E.P., Gregory J.F. (2003). Effect of food fortification on folic acid intake in the United States. Am. J. Clin. Nutr..

[30] Heyssel R.M., Bozian R.C., Darby W.J., Bell M.C. (1966). Vitamin B12 turnover in man. Theassimilation of vitamin B12 from natural foodstuff by man and estimates of minimal daily requirements. Am. J. Clin. Nutr..

[31] Stabler S.P., Allen R.H. (2004). Vitamin B12 deficiency as a worldwide problem. Annu. Rev. Nutr..

[32] Watanabe F. (2007). Vitamin B12 sources and bioavailability. Exp. Biol. Med..

[33] Gilsing A.M., Crowe F.L., Lloyd-Wright Z. (2010). Serum concentrations of Vitamin B12 and folate in British male omnivores, vegetarians and vegans: Results from a cross-sectional analysis of the EPIC-Oxford cohort study. Eur. J. Clin. Nutr..

[34] Herbert V., Ziegler E.E., Filer L.J. (1996). Vitamin B-12. Present Knowledge in Nutrition.

[35] Scalabrino G. (2009). The multi-faceted basis of vitamin B12 (cobalamin) neurotrophism in adult central nervous system: Lessons learned from its deficiency. Prog. Neurobiol..

[36] Carmel R. (2008). How I treat cobalamin (vitamin B12) deficiency. Blood.

[37] Hultdin J., van Guelpen B., Bergh A., Hallmans G., Stattin P. (2005). Plasma folate, Vitamin B12, and homocysteine and prostate cancer risk: A prospective study. Int. J. Cancer.

[38] Heaney R.P., Abrams S., Dawson-Hughes B. (2000). Peak bone mass. Osteoporos. Int..

[39] Barker M.E., Blumsohn A. (2009). Calcium, vitamin D and weight loss. Br. J. Nutr..

[40] Weaver C.M., Coulston A.M., Boushey C. (2008). Osteoporosis: The early years. Nutrition in the Prevention and Treatment of Disease.

[41] Krall E.A., Dawson-Hughes B. (1993). Heritable and life-style determinants of bone mineral density. J. Bone Miner. Res..

[42] Ackerman K.E., Misra M. (2011). Bone Health in Adolescent Athletes with a Focus on Female Athlete Triad. Phys. Sportsmed..

[43] Karaguzel G., Holick M.F. (2010). Diagnosis and treatment of osteopenia. Rev. Endocr. Metab. Disord..

[44] Dawson-Hughes B., Holick M.F., Dawson-Hughes B. (2004). Calcium and vitamin D for bone health in adults. Nutrition and Bone Health.

[45] Touvier J., Winzenrieth R., Johansson H., Roux J.P., Chaintreuil J., Toumi H., Jennane R., Hans D., Lespessailles E. (2015). Fracture discrimination by combined bone mineral density (BMD) and microarchitectural texture analysis. Calcif. Tissue Int..

[46] Looker A.C., Orwoll E.S., Johnston C.C.J., Lindsay R.L., Wahner H.W., Dunn W.L. (1997). Prevalence of low femoral bone density in older U.S. adults from NHANES III. J. Bone Miner. Res..

[47] Haczynski J., Jakimiuk A. (2001). Vertebral fractures: A hidden problem of osteoporosis. Med. Sci. Monit..

[48] Cauley J.A., Thompson D.E., Ensrud K.C., Scott J.C., Black D. (2000). Risk of mortality following clinical fractures. Osteoporos. Int..

[49] Johnell O., Kanis J.A. (2006). An estimate of the worldwide prevalence and disability associated with osteoporotic fractures. Osteoporos. Int..

[50] Kraft K. (2009). Complementary/alternative medicine in the context of prevention of disease and maintenance of health. Prev. Med..

[51] Rondanelli M., Opizzi A., Perna S., Faliva M.A. (2013). Update on nutrients involved in maintaining healthy bone. Endocrinol. Nutr..

[52] Herrmann M., Widmann T., Colaianni G., Colucci S., Zallone A., Herrmann W. (2005). Increased osteoclast activity in the presence of increased homocysteine concentrations. Clin. Chem..

[53] Koh J., Lee Y., Kim Y. (2006). Homocysteine enhances bone resorption by stimulation of osteoclast formation and activity through increased intracellular ROS generation. J. Bone Miner. Res..

[54] Kim D.J., Koh J., Lee O. (2007). Homocysteine enhances apoptosis in human bone marrow stromal cells. Bone.

[55] Liu G., Nellaiappan K., Kagan H.M. (1997). Irreversible inhibition of lysyl oxidase by homocysteinethiolactone and its selenium and oxygen analogues. Implications for homocystinuria. J. Biol. Chem..

[56] Raposo B., Rodriguez C., Martinez-Gonzalez J., Badimon L. (2004). High levels of homocysteine inhibit lysyl oxidase (LOX) and downregulate LOX expression in vascular endothelial cells. Atherosclerosis.

[57] Herrmann M., Schmidt J., Umanskaya N. (2007). Stimulation of osteoclast activity by low B-vitamin concentrations. Bone.

[58] Thaler R., Agsten M., Spitzer S. (2011). Homocysteine suppresses the expression of the collagen cross-linker lysyl oxidase involving IL-6, Fli1, and epigenetic DNA methylation. J. Biol. Chem..

[59] Saito M., Fujii K., Marumo K. (2006). Degree of mineralizationrelated collagen crosslinking in the femoral neck cancellous bone in cases of hip fracture and controls. Calcif. Tissue Int..

[60] Neetu T., Madhavi K., Charu M., Jonathan C.V., Natia Q., Pushpakumar S.B., Naria M., Suresh C.T. (2011). Homocysteine mediated decrease in bone bloodflowand remodeling: Role of Folic Acid. J. Orthop. Res..

[61] Weiss N., Heydrick S., Zhang Y.Y., Bierl C., Cap A., Loscalzo J. (2002). Cellular redox state and endothelial dysfunction in mildly hyperhomocysteinemiccystathionine beta-synthase-deficient mice. Arterioscler. Thromb. Vasc. Biol..

[62] Sanchez-Rodriguez M.A., Ruiz-Ramos M., Correa-Munoz E., Mendoza-Nunez V.M. (2007). Oxidative stress as a risk factor for osteoporosis in elderly Mexicans as characterized by antioxidant enzymes. BMC Musculoskelet. Disord..

[63] Clemens T.L. (2014). Vitamin B12 deficiency and bone health. N. Engl. J. Med..

[64] Roman-Garcia P., Quiros-Gonzalez I., Mottram L., Lieben L., Sharan K., Wangwiwatsin A., Tubio J., Lewis K., Wilkinson D., Santhanam B. (2014). Vitamin B_12_-dependent taurine synthesis regulates growth and bone mass. J. Clin. Investig..

[65] McLean R.R., Jacques P.F., Selhub J. (2004). Homocysteine as a predictive factor for hip fracture in older persons. N. Engl. J. Med..

[66] McKusick V.A. (1966). Heritable Disorders of Connective Tissue.

[67] Lubec B., Fang-Kircher S., Lubec T. (1996). Evidence for McKusick’s hypothesis of deficient collagen crosslinking in patients with homocystinuria. Biochim. Biophys. Acta.

[68] Holstein J.H., Herrmann M., Splett C., Herrmann W., Garcia P., Histing T., Klein M., Kurz K., Siebel T., Pohlemann T. (2011). High bone concentrations of homocysteine are associated with altered bone morphology in humans. Br. J. Nutr..

[69] Vaes B.L.T., Lute C., van der Woning S.P., Piek E., Vermeer J., Blom H.J., Mathers J.C., Müller M., de Groot L.C.P.G.M., Steegenga W.T. (2010). Inhibition of methylation decreases osteoblast differentiation via a non- DNA-dependent methylation mechanism. Bone.

[70] Herrmann M., Tami A., Wildemann B., Wolny M., Wagner A., Schorr H., Taban-Shomal O., Umanskaya N., Ross S., Garcia P. (2009). Hyperhomocysteinemia induces a tissue specific accumulation of homocysteine in bone by collagen binding and adversely affects bone. Bone.

[71] Gjesdal C.G., Vollset S.E., Ueland P.M., Refsum H., Meyer H.E., Tell G.S. (2007). Plasma homocysteine, folate, and vitamin B 12 and the risk of hip fracture: The hordalandhomocysteine study. J. Bone Miner. Res..

[72] Gerdhem P., Ivaska K.K., Isaksson A. (2007). Associations between homocysteine, bone turnover, BMD, mortality, and fracture risk in elderly women. J. Bone Miner. Res..

[73] Perier M.A., Gineyts E., Munoz F., Sornay-Rendu E., Delmas P.D. (2007). Homocysteine and fracture risk in postmenopausal women: The OFELY study. Osteoporos. Int..

[74] Ravaglia G., Forti P., Maioli F. (2005). Folate, but not homocysteine, predicts the risk of fracture in elderly persons. J. Gerontol. A Biol. Sci. Med. Sci..

[75] O’Leary F., Samman S. (2010). Vitamin B12 in health and disease. Nutrients.

[76] Holstein J.H., Herrmann M., Splett C., Herrmann W., Garcia P., Histing T. (2009). Low serum folate and vitamin B-6 are associated with an altered cancellous bone structure in humans. Am. J. Clin. Nutr..

[77] McLean R.R., Jacques P.F., Selhub J. (2008). Plasma B Vitamins, Homocysteine, and their relation with bone loss and hip fracture in elderly men and women. J. Clin. Endocrinol. Metab..

[78] Carmel R., Lau K.H., Baylink D.J., Saxena S., Singer F.R. (1988). Cobalamin and osteoblast-specific proteins. N. Engl. J. Med..

[79] Dhonukshe-Rutten R.A., Pluijm S.M., de Groot L.C., Lips P., Smit J.H., van Staveren W.A. (2005). Homocysteine and vitamin B12 status relate to bone turnover markers, broadband ultrasound attenuation, and fractures in healthy elderly people. J. Bone Miner. Res..

[80] Levasseur R. (2009). Bone tissue and hyperhomocysteinemia. Joint Bone Spine.

[81] Hofman A., van Duijn C.M., Franco O.H., Ikram M.A., Janssen H.L., Klaver C.C., Kuipers E.J., Nijsten T.E., Stricker B.H., Tiemeier H. (2011). The Rotterdam Study: 2012 objectives and design update. Eur. J. Epidemiol..

[82] Yazdanpanah N., Zillikens M.C., Rivadeneira F., de Jong R., Lindemans J., Uitterlinden A.G., Pols H.A., van Meurs J.B. (2007). Effect of dietary B vitamins on BMD and risk of fracture in elderly men and women: The Rotterdam study. Bone.

[83] Reynolds T., Marshall P., Brain A. (1992). Patients with hip fracture may be vitamin B6 deficient. Acta Orthop. Scand..

[84] Gjesdal C.G., Vollset S.E., Ueland P.M. (2006). Plasma total homocysteine level and bone mineral density—The Hordalandhomocysteine Study. Arch Intern. Med..

[85] Cagnacci A., Bagni B., Zini A., Cannoletta M., Generali M., Volpe A. (2008). Relation of folates, vitamin B12 and homocysteine to vertebral bone mineral density change in postmenopausal women. A five-year longitudinal evaluation. Bone.

[86] Sleight P. (2000). The HOPE Study (Heart Outcomes Prevention Evaluation). J. Renin Angiotensin Aldosterone Syst..

[87] Sawka A.M., Ray J.G., Yi Q., Josse R.G., Lonn E. (2007). Randomized clinical trial of homocysteine level lowering therapy and fractures. Arch Intern. Med..

[88] Van Wijngaarden J.P., Dhonukshe-Rutten R.A., van Schoor N.M., van der Velde N., Swart K.M., Enneman A.W., van Dijk S.C., Brouwer-Brolsma E.M., Zillikens M.C., van Meurs J.B. (2011). Rationale and design of the B-PROOF study, a randomized controlled trial on the effect of supplemental intake of Vitamin B12 and folic acid on fracture incidence. BMC Geriatr..

[89] Van Wijngaarden J.P., Swart K.M., Enneman A.W., Dhonukshe-Rutten R.A., van Dijk S.C., Ham A.C., Brouwer-Brolsma E.M., van der Zwaluw N.L., Sohl E., van Meurs J.B. (2014). Effect of daily vitamin B-12 and folic acid supplementation on fracture incidence in elderly individuals with an elevated plasma homocysteine concentration: B-PROOF, a randomized controlled trial. Am. J. Clin. Nutr..

[90] Gommans J., Yi Q., Eikelboom J.W., Hankey G.J., Chen C., Rodgers H. (2013). The effect of homocysteine-lowering with B-vitamins on osteoporotic fractures inpatients with cerebrovascular disease: Substudyof VITATOPS, a randomised placebo-controlled trial. BMC Geriatr..

[91] Hong X., Hsu Y.H., Terwedow H., Tang G., Liu X., Jiang S., Xu X., Xu X. (2007). Association of the methylenetetrahydrofolatereductase C677T polymorphism and fracture risk in Chinese postmenopausal women. Bone.

[92] Riancho J.A., Valero C., Zarrabeitia M.T. (2006). MTHFR polymorphism and bone mineral density: Meta-analysis of published studies. Calcif. Tissue Int..

[93] Green T., McMahon J., Skeaff C. (2007). Lowering homocysteine with B Vitamins has no effect on biomarkers of bone turnover in older persons: A 2-y randomized controlled trial. Am. J. Clin. Nutr..

[94] Sato Y., Honda Y., Iwamoto J., Kanoko T., Satoh K. (2005). Effect of folate and mecobalamin on hip fractures in patients with stroke: A randomized controlled trial. JAMA.

[95] Malinow M.R., Bostom A.G., Krauss R.M. (1999). Homocyst(e)ine, diet, and cardiovascular diseases: A statement for healthcare professionals from the Nutrition Committee, American Heart Association. Circulation.

[96] Frosst P., Blom H.J., Milos R., Goyette P., Sheppard C.A., Matthews R.G., Boers G.J., den Heijer M., Kluijtmans L.A., van den Heuvel L.P. (1995). A candidate genetic risk factor for vascular disease: A common mutation in methylenetetrahydrofolatereductase. Nat. Genet..

[97] Molloy A., Daly S., Mills J. (1997). Thermolabile variant of 5,10-methylenetetrahydrofolate reductase associated with low red-cell folates: Implications for folate intake recommendations. Lancet.

[98] Casas J.P., Bautista L.E., Smeeth L., Sharma P., Hingorani A.D. (2005). Homocysteine and stroke: Evidence on a causal link from mendelianrandomisation. Lancet.

[99] Reilly R., McNulty H., Pentieva K., Strain J.J., Ward M. (2014). MTHFR 677TT genotype and disease risk: Is there a modulating role for B-Vitamins?. Proc. Nutr. Soc..

[100] Yamada K., Chen Z., Rozen R. (2001). Effects of common polymorphisms on the properties of recombinant human methylenetetrahydrofolatereductase. Proc. Natl. Acad. Sci. USA.

[101] Macdonald H.M., McGuigan F.E., Fraser W.D., New S.A., Ralston S.H., Reid D.M. (2004). Methylenetetrahydrofolatereductase polymorphism interacts with riboflavin intake to influence bone mineral density. Bone.

[102] McLean R.R., Karasik D., Selhub J. (2004). Association of a common polymorphism in the methylenetetrahydrofolatereductase (MTHFR) gene with bone phenotypes depends on plasma folate status. J. Bone Miner. Res..

[103] Abrahamsen B., Madsen J.S., Tofteng C.L., Stilgren L., Bladbjerg E.M., Kristensen S.R, Brixen X., Mosekilde L. (2005). Are effects of MTHFR (C677T) genotype on BMD confined to women with low folate and riboflavin intake? Analysis of food records from the Danish osteoporosis prevention study. Bone.

